# Continuous Professional Oral Health Care Intervention Improves Severe Aspiration Pneumonia

**DOI:** 10.1155/2019/4945921

**Published:** 2019-12-16

**Authors:** Wakako Nawata, Yojiro Umezaki, Masahiro Yamaguchi, Masato Nakajima, Michiko Makino, Masahiro Yoneda, Takao Hirofuji, Takafumi Yamano, Hiroaki Ooboshi, Hiromitsu Morita

**Affiliations:** ^1^The Center for Visiting Dental Service, Fukuoka Dental College Medical and Dental General Hospital, Fukuoka, Japan; ^2^Division of Dental Hygiene, Fukuoka Dental College Medical and Dental General Hospital, Fukuoka, Japan; ^3^Section of Geriatric Dentistry, Department of General Dentistry, Fukuoka Dental College, Fukuoka, Japan; ^4^Section of General Dentistry, Department of General Dentistry, Fukuoka Dental College, Fukuoka, Japan; ^5^Section of Otorhinolaryngology, Department of General Medicine, Fukuoka Dental College, Fukuoka, Japan; ^6^Section of Internal Medicine, Department of General Medicine, Fukuoka Dental College, Fukuoka, Japan

## Abstract

Professional oral health care (POHC) is known to prevent aspiration pneumonia in patients with dysphagia and/or those at the perioperative stage of surgery. However, the effect of POHC on patients suffering from aspiration pneumonia remains unknown. Here, we report a case where continual POHC intervention improved severe aspiration pneumonia. A 74-year-old male patient with a brain infarction suffered from severe aspiration pneumonia (PSI: IV, A-DROP: 3) complicated by vascular dementia and severe dysphagia. Because an antimicrobial approach following the treatment guidelines for pneumonia was not effective, we started a POHC intervention to improve his poor oral condition at the request of the attending doctor and the patient's family. The severe pneumonia markedly improved after continual POHC by the dental team. This case suggests that continual POHC intervention by a dental hygienist may improve severe aspiration pneumonia.

## 1. Introduction

Professional oral health care (POHC) prevents aspiration pneumonia caused by aspiration in older adults, especially when it is associated with dysphagia [1–6]. However, there is little evidence that POHC intervention after illness improves severe aspiration pneumonia.

Here, we report a case in which continual POHC intervention improved severe aspiration pneumonia caused by *Klebsiella pneumoniae* and/or multidrug-resistant *Pseudomonas aeruginosa* (MDRP) in a patient with a brain infarction complicated by vascular dementia and dysphagia.

## 2. Case Report

**Figure 1:**
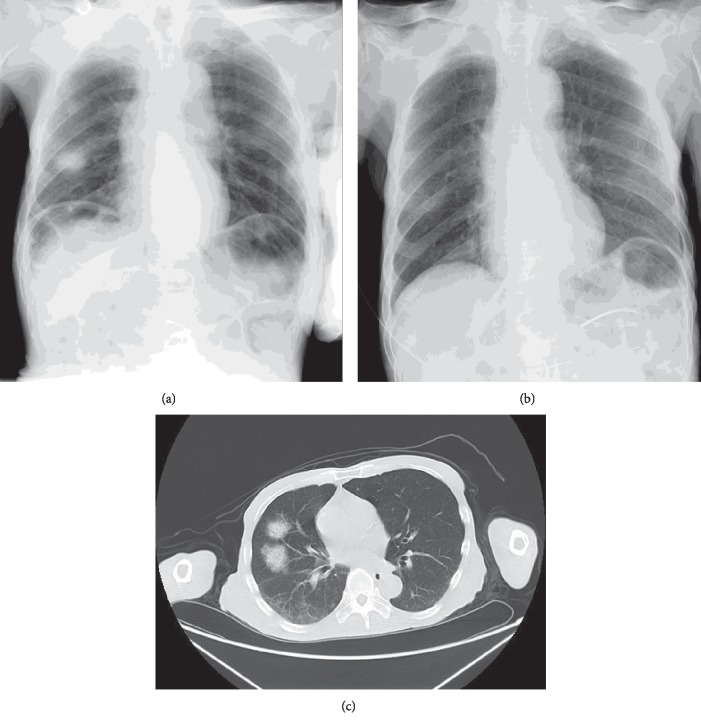
Plain X-ray and CT image. Reticular shadow with ground-glass opacity was observed in the medial lobe of the right lung in the plain X-ray (a) and CT (c) on abnormalities disappeared 45 days later (b).

A 74-year-old male residing in a nursing home was hospitalized with a diagnosis of aspiration pneumonia. *Klebsiella pneumoniae* was detected by sputum culture. He was treated with tazobactam/piperacillin (TAZ/PIPC) following the guidelines for the treatment of respiratory infectious diseases including community-acquired pneumonia (CAP) in Japan. The patient's medical history was as follows: multiple cerebral infarctions, hypertension, diabetes mellitus, and chronic kidney disease. Vascular dementia with parkinsonism and severe dysphagia were also diagnosed. He took nutrition by nasoenteric feeding, and his consciousness was interpreted as drowsiness (Japan coma scale (JCS): II-20). The severity of the pneumonia was moderate (pneumonia severity index (PSI): II and CAP severity index (A-DROP): 2). Plain X-ray and computed tomography (CT) images are shown in Figure 1. Written informed consent was obtained from the patient's family for publication of this case report following the Ethical Guidelines of Fukuoka Dental College, and this case study was approved by the ethical committee of Fukuoka Dental College (#370).

**Figure 2:**
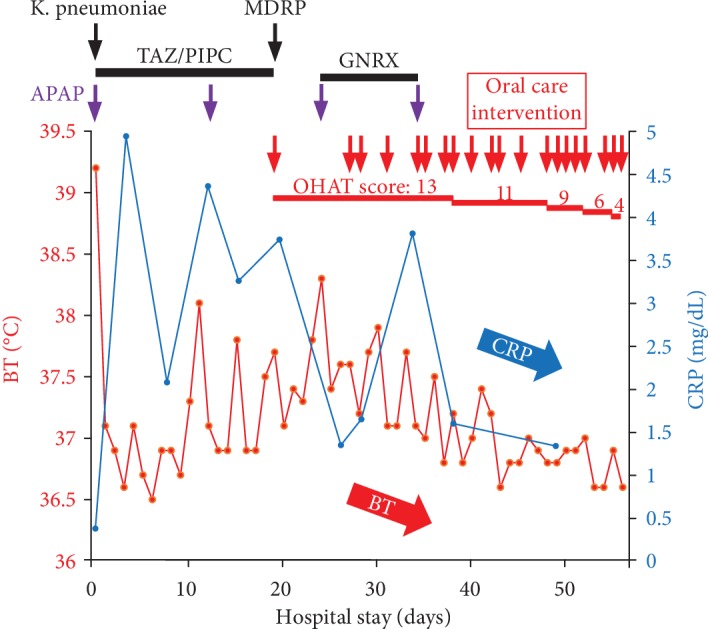
Time course of body temperature (BT) and C-reactive protein (CRP). BT and CRP are indicated by red closed circles and a solid line and blue closed circles and a solid line, respectively. Detection of bacteria by sputum culture, administration of acetaminophen (APAP), and professional oral health care intervention are indicated by black, purple, and red arrows, respectively. Treatment durations of tazobactam/piperacillin (TAZ/PIPC) and garenoxacin (GNRX) are indicated by solid bars. Oral Health Assessment Tool (OHAT) scores, assessed by the patient's oral condition at each timepoint, are indicated by red colored and red underlined text in the upper part of the figure.

The patient transiently recovered from the fever, and the elevated C-reactive protein (CRP) levels returned to normal after TAZ/PIPC and acetaminophen therapy. However, exacerbation of the fever and elevated CRP levels occurred within a couple of days. MDRP was detected in his sputum culture (Figure 2) and the severity of the pneumonia increased (PSI: IV and A-DROP: 3). Because the MDRP isolate was only sensitive to fluoroquinolone and cephem, garenoxacin was administered. However, the patient's fever and CRP levels did not improve. Because liver damage was then observed (aspartate transaminase (AST): 980 IU/L, alanine transaminase (ALT): 733 IU/L, and gamma-glutamyl transpeptidase (*γ*-GTP): 99 IU/L), garenoxacin was discontinued (Figure 2).

**Figure 3:**
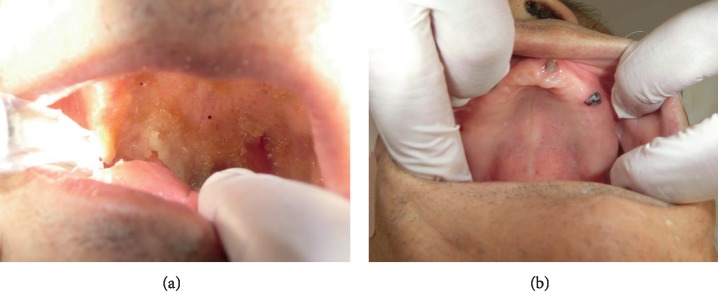
Oral condition at (a) initial dental examination and (b) after receiving professional oral health care.

POHC was requested by the attending physician and the patient's family to improve his oral condition, along with physical rehabilitation. His intraoral condition was poor and quite dry. He had only three remaining natural teeth, which were all missing their crowns (13, 21, and 23; Figure 3). Multiple membranous substances were found to be adhering to the surface of the tongue, palate, and buccal mucosa at the initial oral examination (Figure 3(a), Table 1, Oral Health Assessment Tool (OHAT) score: 13). We performed twice daily POHC to clean multiple membranous substances carefully using disposable sponge brushes and moisturizing gel (Refrecare®, EN Otsuka Pharmaceutical Co., Ltd., Tokyo, Japan), which also provided moisture for stabilizing the oral mucosa and tongue dorsum. In the early stages of POHC intervention, the patient's oral condition worsened and became dry 1 day after POHC. An oral-candidiasis-like redness on his oral mucosa and the dorsum of the tongue was also observed after removal of multiple membranous substances. The patient seemed to feel pain when we touched his oral mucosa despite our gentle procedure using moisturizing gel. However, our persistent POHC gradually became effective in achieving a healthy oral condition 2 weeks after starting the POHC intervention. Additionally, the patient ceased expressing pain vocally and through facial expression during POHC, and the redness of the oral mucosa totally disappeared (Figures 2 and 3, Table 1). Following recovery of his oral health, the patient's fever subsided and CRP levels returned to within the normal range. The X-ray abnormality and his consciousness (JCS: I-3) also improved (Figures 1(b) and 2). Finally, he was moved to another hospital for recuperation almost 1 month after starting POHC.

## 3. Discussion

Here, we report a case in which the patient recovered from severe aspiration pneumonia after continual POHC intervention. The main cause of pneumonia was thought to be aspiration of oral bacteria under poor oral conditions complicated by dysphagia. This case suggests that, in addition to antimicrobial treatment, the POHC intervention should have commenced at the initial stages of pneumonia. In fact, pneumonia did not improve until the POHC intervention began. However, persistent POHC intervention effectively contributed to improving the OHAT score (from 13 to 4) as well as lessening the severity of the pneumonia [7]. The Japanese guidelines for respiratory infectious diseases and CAP treatment also recommend that when antibiotics are not effective, physicians should consider systemic etiologic factors, including the presence of antibiotic-resistant bacteria, although the side effects of the antibiotics themselves can also include a rising fever and an elevated CRP level [8–10]. Another possible mechanism of recurrent inflammation observed in this case is repetitive aspiration of oral pathogens. Our results suggest that POHC involving the mechanical removal of oral pathogens may prevent the exacerbation of aspiration pneumonia in patients with dysphagia, notwithstanding the effects of antibiotics. Interestingly, several reports have shown evidence that *Candida albicans*, a commensal and opportunistic pathogen, forms a biofilm when cocultured with *K. pneumoniae* and other oral bacteria as pathogens of bacterial pneumonia [11]. Biofilms are often intrinsically resistant to conventional antifungal and antibiotic therapeutics. POHC can reduce the number of oral and oropharyngeal bacteria by mechanically removing bacterial biofilms, including respiratory pathogens [12]. We did not perform a culture test for the diagnosis of oral candidiasis in this case, although some clinical features of oral candidiasis, such as redness of the oral mucosa and sore mouth, were observed [13, 14]. It is therefore reasonable to presume that the antibiotic treatment was ineffective, but mechanical POHC was effective, in the recovery from aspiration pneumonia in this case.

Currently, POHC is recommended for the prevention of fever and aspiration pneumonia in older adults; however, no clear evidence exists that it can suppress the exacerbation of aspiration pneumonia [1–6]. There is some evidence that POHC can contribute to a better quality of life by improving oral complications such as halitosis, xerostomia, and oral infectious diseases in the terminal stages of systemic diseases [15]. Moreover, mechanical stimulation of the oral mucosa by POHC leads to brain stimulation and improvements in dysphagia, latency time of the swallowing response, and the cough reflex [16–18]. While further study is needed, POHC may become a supportive therapy for patients with severe aspiration pneumonia even if they are in the terminal stages of disease.

In conclusion, POHC may contribute to preventing both the occurrence and exacerbation of aspiration pneumonia by improving patients' oral condition.

## Figures and Tables

**Table 1:** Evaluation results by Oral Health Assessment Tool: category scores at initial day and last day of oral care.

Category	0 = healthy	1 = changes	2 = unhealthy	Category scores (initial day)	Category scores (last day)
Lips	Smooth, pink, moist	Dry, chapped, or red at corners	Swelling or lump, white/red/ulcerated patch; bleeding/ulcerated at corners	1	0
Tongue	Normal, moist roughness, pink	Patchy, fissured, red, coated	Patch that is red and/or white, ulcerated, swollen	1	0
Gums and tissues	Pink, moist, smooth, no bleeding	Dry, shiny, rough, red, swollen, one ulcer/sore spot under dentures	Swollen, bleeding, ulcers, white/red patches, generalized redness under dentures	1	0
Saliva	Moist tissues, watery and free flowing saliva	Dry, sticky tissues, little saliva present, resident thinks they have a dry mouth	Tissues parched and red, very little/no saliva present, saliva is thick, resident thinks they have a dry mouth	2	0
Natural teeth Yes/no	No decayed or broken teeth/roots	1-3 decayed or broken teeth/roots or very worn down teeth	4+ decayed or broken teeth/roots, or very worn down teeth, or less than 4 teeth	2	2
Dentures Yes/no	No broken areas or teeth, dentures regularly worn, and named	1 broken area/tooth or dentures only worn for 1-2 h daily, or dentures not named, or loose	More than 1 broken area/tooth, denture missing or not worn, loose and needs denture adhesive, or not named	2	2
Oral cleanliness	Clean and no food particles or tartar in mouth or dentures	Food particles/tartar/plaque in 1-2 areas of the mouth or on small area of dentures or halitosis (bad breath)	Food particles/tartar/plaque in most areas of the mouth or on most of the dentures or severe halitosis (bad breath)	2	0
Dental pain	No behavioural, verbal, or physical signs of dental pain	Verbal and/or behavioural signs of pain such as pulling at face, chewing lips, not eating, aggression	Physical pain signs (swelling of cheek or gum, broken teeth, ulcers), as well as verbal and/or behavioural signs (pulling at face, not eating, aggression)	2	0
Total score (maximum: 16)	—	—	—	13	4
